# Stimulation of histamine H_1_-receptors produces a positive inotropic effect in the human atrium

**DOI:** 10.1007/s00210-024-03735-y

**Published:** 2024-12-27

**Authors:** Thanh Hoai Pham, Lina Maria Rayo Abella, Katarina Hadova, Jan Klimas, Stefan Dhein, Steffen Pockes, Jonas Manfred Albert Schlicht, Britt Hofmann, Uwe Kirchhefer, Joachim Neumann, Ulrich Gergs

**Affiliations:** 1 Institute for Pharmacology and Toxicology, Medical Faculty, Martin Luther University Halle-Wittenberg, Magdeburger Straße 4, D-06097 Halle (Saale), Germany; 2 Department of Pharmacology and Toxicology, Faculty of Pharmacy, Comenius University Bratislava, Odbojárov 10, SK-832 32 Bratislava, Slovak Republic; 3 Rudolf‑Boehm Institute for Pharmacology and Toxicology, University Leipzig, Härtelstraße 16‑18, D‑04107 Leipzig, Germany; 4 Institute of Pharmacy, University of Regensburg, Universitätsstraße 31, 93053 Regensburg, Germany; 5 Department of Cardiac Surgery, Mid-German Heart Centre, University Hospital Halle, Ernst-Grube-Str. 40, D‑06097 Halle (Saale), Germany; 6 Institute for Pharmacology and Toxicology, Medical Faculty, University Münster, Domagkstraße 12, D-48149 Münster, Germany

**Keywords:** Human histamine H_1_-receptors, Transgenic mouse atrium, Human atrium, Force of contraction

## Abstract

There is a controversy whether histamine H_1_-receptor activation raises or lowers or does not affect contractility in the human heart. Therefore, we studied stimulation of H_1_-receptors in isolated electrically stimulated (one beat per second) human atrial preparations (HAP). For comparison, we measured force of contraction in left atrial preparations (LA) from mice with overexpression of the histamine H_1_-receptor in the heart (H_1_-TG). We detected the messenger ribonucleic acid (mRNA) expression of human histamine H_1_-receptors in HAP. In LA from H_1_-TG, each cumulatively applied concentration of histamine and a dual H_1_/H_2_-agonist called 2-(2-thiazolyl)-ethylamine (ThEA) caused a time-dependent initial negative inotropic effect followed over time by a lasting positive inotropic effect. Both effects were concentration-dependent in LA from H_1_-TG. After 100 µM cimetidine, 10 µM histamine exercised a positive inotropic effect in HAP that was diminished by 10 µM mepyramine, an H_1_-receptor antagonist. The concentrations of mepyramine and cimetidine used here are based on the work of others and our own work (e.g., Guo et al. J Cardiovasc Pharmacol. 6:1210-5 1984, Rayo Abella et al. J Pharmacol Exp Ther. 389:174-185 2024). Similarly, we observed that ThEA (10 µM, 30 µM, 100 µM cumulatively applied) induced a concentration- and time-dependent positive inotropic effect in HAP. In HAP, we detected never negative inotropic effects to either histamine or ThEA. The positive inotropic effects to ThEA in HAP were reduced by mepyramine. The positive inotropic effects of ThEA in LA from H_1_-TG and in HAP were not accompanied by reductions in the time of tension relaxation. We conclude that stimulation of histamine H_1_-receptors only increases and does not decrease force of contraction in the HAP in our patients.

**Supplementary Information:**

The online version contains supplementary material available at 10.1007/s00210-024-03735-y.

## Introduction

In the heart, all currently known histamine receptor subtypes have been detected on RNA-level and/or protein-level (review: Neumann et al. 2021a). However, histamine receptor function shows species differences, but even regional differences (review: Neumann et al. 2021a, Neumann et al. 2023). In the mouse heart, histamine does not augment contractility (Gergs et al. 2019, 2020, 2021a, 2021b, Neumann et al. 2021b, Neumann et al. 2021c, Neumann et al. 2021d). Likewise, in the murine and feline heart, histamine releases noradrenaline from cardiac stores (Bartlet 1963, Flacke et al. 1967, Dai 1976, Laher und McNeill 1980a, 1980b, Wellner-Kienitz et al. 2003).

**Fig. 1:**
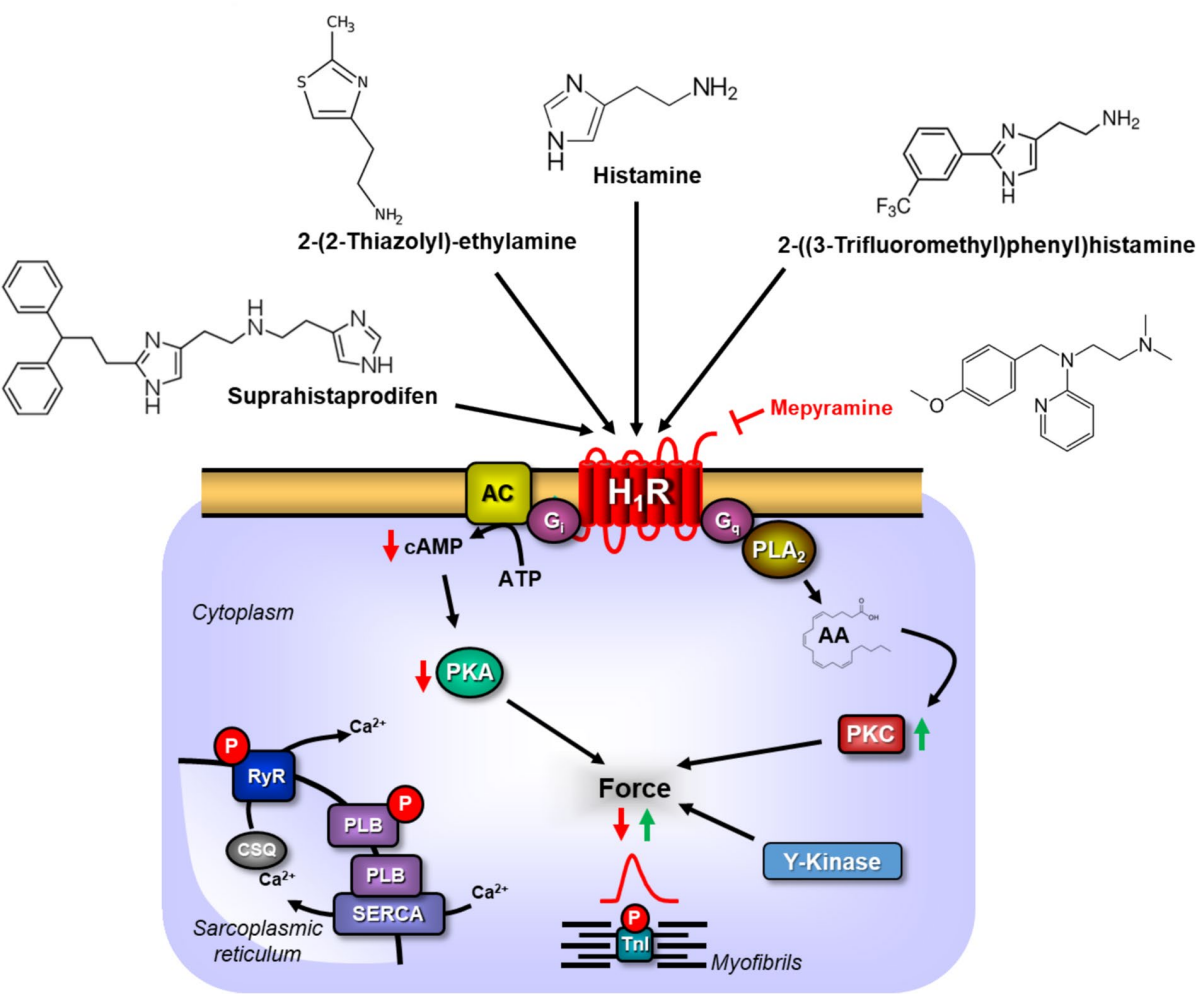
Potential mechanism(s) of action of histamine H_1_-receptor in cardiomyocytes and chemical structures of agonists and antagonist. Histamine H_1_-receptors (H_1_R) are located in the sarcolemma of human cardiomyocytes. They are stimulated by histamine, 2-(2-thiazolyl)-ethylamine (ThEA), 2-((3-trifluoromethyl)phenyl)histamine, and suprahistaprodifen. They are antagonized by mepyramine. They may activate phospholipase A_2_ (PLA_2_) and this may via proteinkinase C increase force of contraction. Alternatively, histamine H_1_-receptor may inhibit the activity of adenylyl cyclases (AC), which may decrease intracellular cAMP and the activity of cAMP-dependent protein kinase A (PKA), and thus decrease force of contraction

In humans, histamine H_1_-receptors (Fig. 1) were identified in atrium and ventricle (antibody studies and mRNA expression: Matsuda et al. 2004). The functional role of histamine H_1_-receptors for inotropy in the human atrium warrants more investigations. Some suggested that like in the guinea pig, a negative chronotropic effect of histamine H_1_-receptors in the human sinus node and a negative dromotropic effect of H_1_-receptor stimulation in the human atrioventricular node might exist (Genovese et al. 1988). Activation of H_1_-receptors was reported to increase or decrease cardiac contractility (Genovese et al. 1988, Zerkowski et al. 1993, Sanders et al. 1996). We have established transgenic mice with cardiac overexpression of human histamine H_1_-receptors (H_1_-TG) to re-evaluate this situation (Rayo Abella et al. 2022). We had before established mice with overexpression of human histamine H_2_-receptors (H_2_-TG). In these mice (H_2_-TG), histamine augmented the contractility of the heart in vitro and in vivo (Gergs et al. 2019, 2020, 2021a, Neumann et al. 2021b,c,d,e). In contrast, in H_1_-TG, histamine led in left atrial preparations a short-lived negative inotropic effect followed by a prolonged positive inotropic effect (Rayo Abella et al. 2022, 2024).

We used in the present communication pharmacological tools namely receptor agonists and antagonists to differentiate receptors acting on inotropy in the human atrium. In order to confirm that we used the proper pharmacological agonists and antagonists in the human atrium, we also tested the effect of histamine in H_1_-TG on force of contraction using the same experimental conditions as in HAP. Mice have the advantage that no inotropically functional histamine receptors are expressed in their hearts. In other words, in LA, added histamine neither increased nor decreased force of contraction (Gergs et al. 2019). Thus, we tested in the present paper the hypothesis that histamine would either increase or decrease force of contraction via histamine H_1_-receptors in HAP.

## Materials and methods

### Generation of transgenic mice

This has been reported before (Rayo Abella et al. 2024). In brief, we made a construct containing the full length cDNA of the human histamine H_1_-receptor under the control of the heart-specific α-myosin heavy chain promoter to obtain transgenic mice. They were kept in a CD-1 background. These mice of both genders were used for experiments when they aged about 100 days.

### Contractile studies in mice

In brief, mice were sacrificed, whole hearts were excised. Then in a petri dish, right or left auricular preparations were dissected and placed into an organ bath (Neumann et al. 1998, Gergs et al. 2019). The bathing solution of the organ baths contained in mM are as follows: 119.8 NaCI, 5.4 KCI, 1.8 CaCl_2_, 1.05 MgCl_2_, 0.42 NaH_2_PO_4_, 22.6 NaHCO_3_, 0.05 Na_2_EDTA, 0.28 ascorbic acid, and 5.05 glucose. The solution was continuously bubbled with 95% O_2_ and 5% CO_2_ and kept at 37°C and pH 7.4 (Neumann et al. 2021e). In contract, left auricles were electrically stimulated with rectangular impulses from a Grass SD9B stimulator (Ohio, USA). The impulses last for 5-ms duration and were 10% voltage above threshold with 1 Hz. The drug application was as follows. After equilibration was reached, propranolol, histamine, ThEA, suprahistaprodifen, or 2-((3-trifluoromethyl)phenyl)histamine were cumulatively added to left atrial preparations to establish concentration–response curves. Then, where indicated, mepyramine was applied to the preparations. In some preparations, indicated in the legends, first cimetidine or cocaine were added before ThEA or histamine or suprahistaprodifen were given.

### Contractile studies on human preparations

HAP were prepared and mounted in the laboratory and were under isometric conditions stimulated with rectangular impulses from a Grass SD9B stimulator (Ohio, USA) with 5-ms duration and 10% voltage above threshold with 1 Hz. The samples were obtained from 15 male patients and 5 female patients, aged 47–81 years. Drug therapy included metoprolol, furosemide, apixaban, statins, and acetyl salicylic acid. Patients suffered from two or three vessel coronary heart disease and comorbidities including obesity, diabetes type 2, and hypertension. Our methods used for atrial contraction studies in human samples have been previously published and were not altered in this study (Boknik et al. 2018, Neumann et al. 2021c, d, e). In some preparations, a complete concentration response curve to histamine alone was generated. In other samples, first or finally mepyramine or cimetidine were applied and thereafter a complete concentration response curve to histamine alone was generated. For details, please see figure legends and original recordings.

### Polymerase chain reaction

Frozen human atrial preparations were pulverized mechanically in liquid nitrogen and were processed by acid phenol–guanidinium thiocyanate–chloroform extraction (TRI Reagent®, Sigma-Aldrich, St. Louis, MO, USA) according to the manufacturer’s instructions. RNA was precipitated from aqueous phase by isopropanol and washed twice with ethanol. Quality of RNA was tested by electrophoresis in 2% agarose gel (Agarose, Sigma-Aldrich, USA). Intact RNA samples were subjected to reverse transcription using High-capacity cDNA Reverse Transcription Kit with RNAse inhibitors (Applied Biosystems, Grand Island, NY, USA). Quantitative real-time polymerase chain reaction (RT-qPCR) analysis was performed on QuantStudio™ 3 Real-Time PCR System (Thermo Fisher Scientific, USA) using SYBR™ Select Master Mix (Thermo Fisher Scientific, USA). Expression of histamine H_1_-receptor in human atrial preparations was determined using gene-specific primers (forward: ATCCCCAGTTGTCTTCAGCC; reverse: CCGGTTGACGGCTACATAGT). The reference gene hypoxanthine phosphoribosyltransferase 1 (HPRT1) was assessed using forward: CCTGGCGTCGTGATTAGTGA and reverse CGAGCAAGACGTTCAGTCCT primers. The primers were designed using Primer-BLAST (Ye et al. 2012). The genes with resulting Cq values higher than 35 (Cq>35) were considered not expressed in the tissue.

### Synthesis of suprahistaprodifen

The suprahistaprodifen was synthesized as published elsewhere (Elz et al. 2000, Schlicker et al. 2001, Menghin et al. 2003).

### Western blotting

Samples were homogenized and protein concentration was measured. Then samples were loaded onto commercial gels, subjected to electrophoresis and transferred to nitrocellulose membranes. Nitrocellulose membranes were cut and incubated with primary and secondary antibodies. Signals were quantified as published recently (Gergs et al. 2024). The studied antibody against histamine H_1_-receptors was from antibodies-online (ABIN719606 (Al12043764), Aachen, Germany; diluted 1:500). As negative controls, we used identically treated heart from knockout mice kindly provided by Dr. Michael Mederos y Schnitzler, Institute for Pharmacology and Toxicology, Ludwig-Maximilians University, Munich, Germany.

### Data analysis

Data shown are means ± standard error of the mean. Statistical significance was estimated using Student’s *t*-test or the analysis of variance followed by Bonferroni’s *t*-test or paired* t*-tests as we regarded as appropriate. See figure legends for details. A *p*-value < 0.05 was considered to be significant.

### Drugs and materials

The drugs isoprenaline hydrochloride, histamine dihydrochloride, cimetidine, and 2-((3-trifluoromethyl)phenyl)histamine were purchased from Sigma-Aldrich (Darmstadt, Germany). 2-(2-Thiazolyl)-ethylamine (ThEA) was purchased from BLD Pharmatech GmbH (Reinbeck, Germany) and mepyramine maleate from Bio-Techne GmbH (Wiesbaden, Germany). All other chemicals were of the highest purity grade commercially available. Deionized water was used throughout the experiments. Stock solutions were prepared fresh daily.

## Results

### PCR

**Fig. 2:**
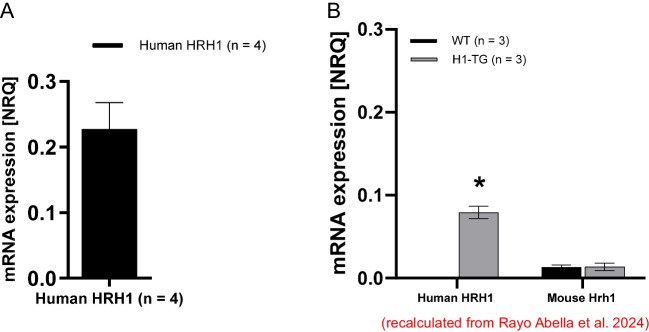
PCR. **A** The relative expression of the target gene human histamine H_1_-receptors (HRH1) in the human right atria presented by NRQ data (normalized relative quantity). Data are normalized to human housekeeping HPRT1 gene. Mean ± SD; *n* = 4. **B** Relative expression of the mRNA of exogenous transgenic histamine H_1_-receptors (HRH1) and endogenous mouse histamine H_1_-receptors (Hrh1) in murine cardiac tissue. The expressions are presented by NRQ data, normalized to mouse housekeeping Hprt1 gene. Data are presented as mean ± SD; *n* = 3 per group. Statistical significance was estimated using Student’s *t*-test; *p < 0.05 versus WT. The data clearly present no expression of exogenous HRH1 gene in WT mice in contrast to endogenous Hrh1 gene. Data in **B** were recalculated from Rayo Abella et al. (2024) and are presented here to facilitate comparison with **A**

Using primers specific for the human histamine H_1_-receptor, we detected expression of the mRNA in the human atrium (Fig. 2A). The purpose of this experiment was to show that the mRNA of the receptor is present in the human atrium. Moreover, we have shown recently that the human histamine H_1_-receptor is present at the mRNA level using in situ hybridization and using RT-PCR in the heart of the H_1_-TG (Fig. 2B: recalculated from Rayo Abella et al. 2024). Hence, the human histamine H_1_-receptor is present in H_1_-TG and in human atrium. However, it is unclear whether there is a common housekeeping gene which we could use for direct comparison between H_1_-TG and human atrium. Regrettably, the primers we used here for the housekeeping gene HPRT1 did not generate specific products in mouse atrium (data not shown), and therefore the relative values of mRNA for the human histamine H_1_-receptor cannot be compared between H_1_-TG and human atrium from our data.

### Western blotting

We detected a signal for the histamine H_1_-receptors in human atrium at the expected molecular weight. However, this signal was also present in H_1_-deficient mice. Hence, the antibody is probably not useful to quantify the amount of histamine H_1_-receptors (Supplementary Data 1; Møller et al. 2016).

### Contraction in the left atrium of H_1_-TG

**Fig. 3:**
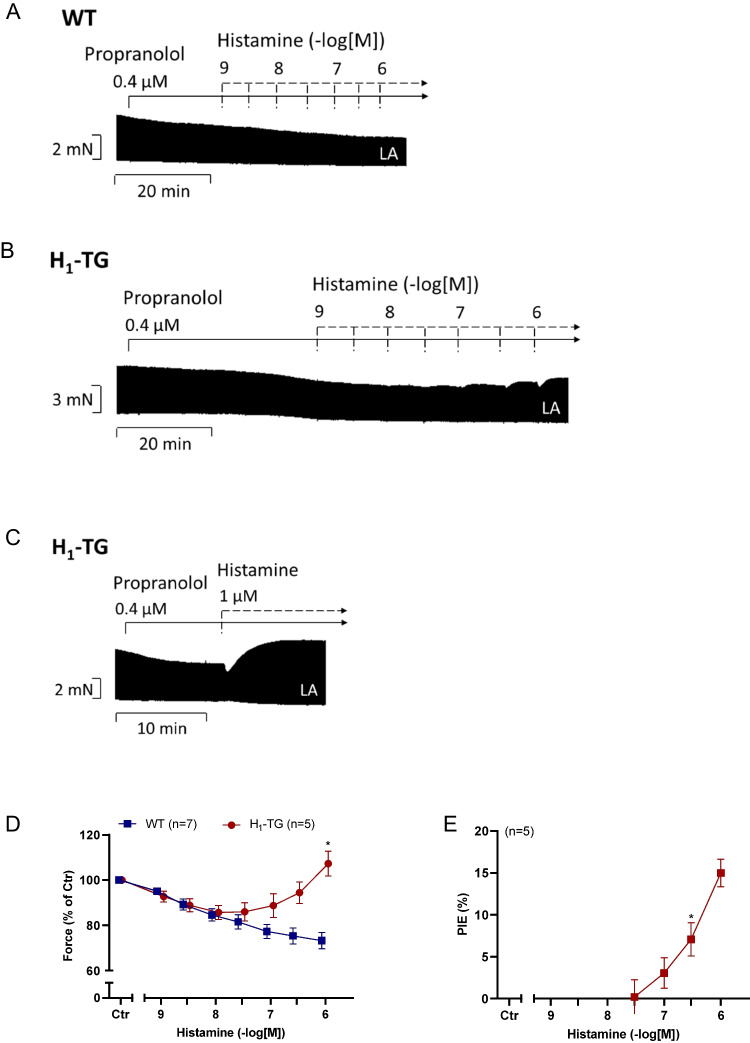
Effects of histamine in left atrial preparations of WT and H_1_-TG mice. Histamine induced time- and concentration-dependently first a negative inotropic effect followed by a positive inotropic effect in atria from mice with heart specific overexpression of the human histamine H_1_-receptor (H_1_-TG). Original recording of force of contraction in isolated electrically stimulated (1 Hz) left atrial (LA) preparation from wild-type mice (WT, **A**) and from H_1_-TG in the presence of 0.4 µM propranolol and increasing concentrations of histamine (Figure 3B) or a single histamine concentration (Figure 3C). Horizontal bar indicate time axis in minutes (min) and ordinate force of contraction in milli Newton (mN). **D** Force of contraction in % of pre-drug value. **E** Percentage of positive inotropic effect (PIE) related to pre-drug value. Abscissae in **D** and **E** indicate concentrations of histamine in negative decadic molar concentrations. Statistical significance was estimated using Student’s *t*-test. *first significant difference (p<0.05) vs. 30 nM histamine. Numbers in brackets indicate number of experiments. Ctr, pre-drug value

Any positive inotropic effect of histamine is lacking in WT (Fig. 3A, D) as reported before by us (Gergs et al. 2019, Neumann et al. 2021a, 2022). We have interpreted this finding to indicate low expression of the histamine H_1_- and H_2_-receptor on protein level and/or defective coupling to inotropic pathways in WT heart. In contrast, histamine exerted a transient negative inotropic effect and a sustained positive inotropic effect in left atrial preparations from H_1_-TG (Fig. 3B, C and D, Rayo Abella et al. 2024). Here we performed novel experiments and plotted the positive inotropic effects of histamine on left atrial preparations of H_1_-TG in Fig. 3E. At 0.3 µM histamine, a PIE reached significance. This indicates that we can detect under our experimental conditions a PIE in H_1_-TG but do not detect any PIE in left atrial preparations from WT (Fig. 3A and D). This suggests that positive inotropic effects of histamine are mediated via cardiac histamine H_1_-receptors under our experimental conditions.

**Fig. 4:**
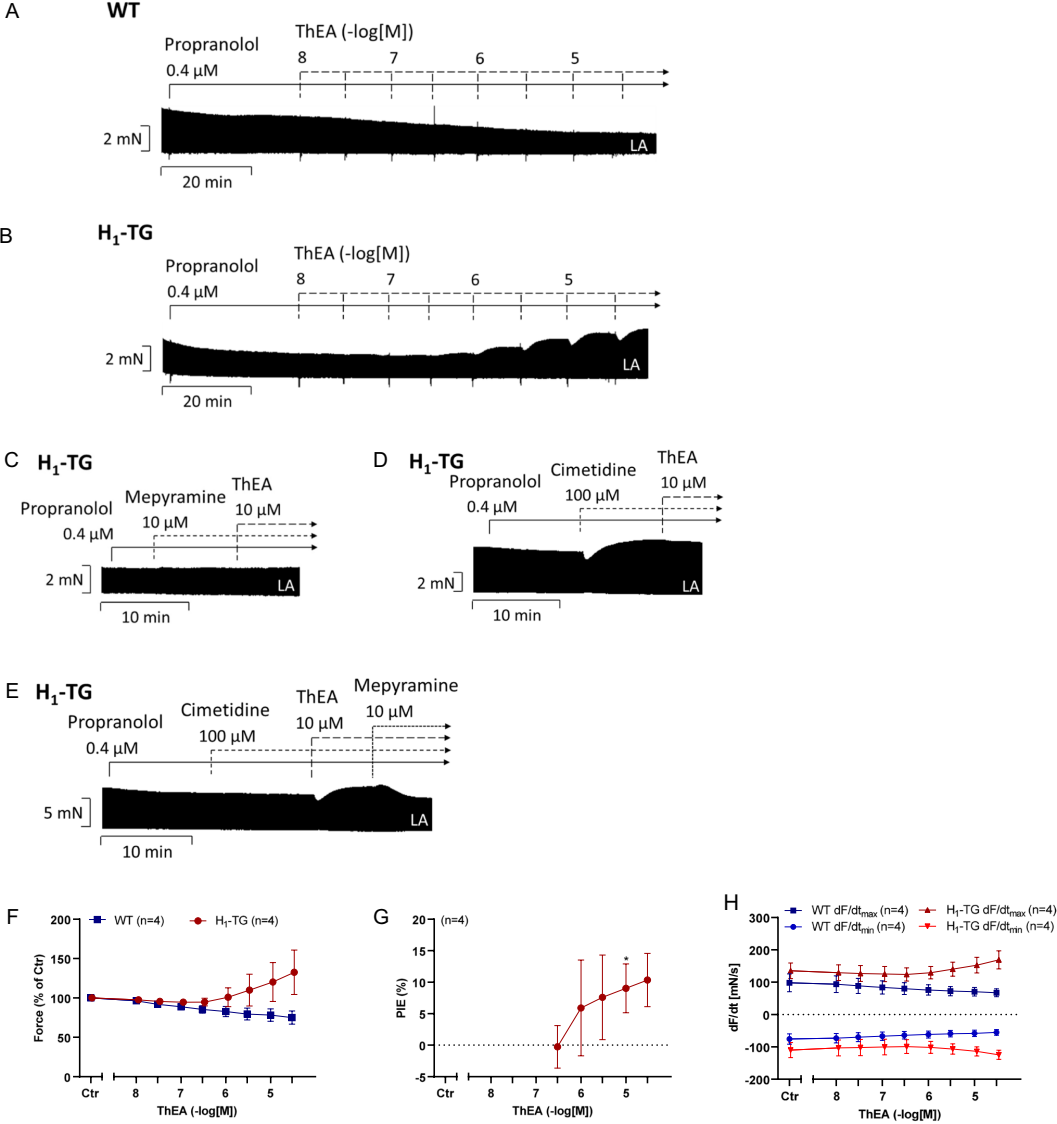
Effects of ThEA in left atrial preparations of WT and H_1_-TG mice. ThEA induced time- and concentration-dependently first a negative inotropic effect followed by a positive inotropic effect in atria from mice with heart specific overexpression of the human histamine H_1_-receptor (H_1_-TG). The biphasic effect of ThEA in H_1_-TG mice could be abolished by the additional presence of the histamine H_1_-receptor antagonist mepyramine, whereas the histamine H_2_-receptor antagonist cimetidine showed no effects on the inotropy of ThEA in H_1_-TG mice. Original recording of force of contraction in isolated electrically stimulated (1 Hz) left atrial (LA) preparations from wild-type mice (WT, **A**) and from H_1_-TG in the presence of 0.4 µM propranolol and increasing concentrations of ThEA (**B**) or 10 µM ThEA with the additional presence of the antagonists mepyramine (10 µM; **C**) or cimetidine (100 µM; **D**) or both in combination (**E**). Horizontal bars in **A**, **B**, **C**, **D**, and **E** indicate time bar in minutes (min). **F** Force of contraction in % of pre-drug value. **G** Positive inotropic effect (PIE) of ThEA in % of pre-drug value. **H** Rate of tension development (dF/dt_max_) and rate of tension relaxation (dF/dt_min_). Ordinates in **A**, **B**, **C**, **D**, and **E**: Force of contraction in milli Newton (mN). Ordinate in **H** is the rate of contraction and rate of relaxation in mN per second (mN/s). Abscissae in **F**, **G**, and **H** indicate concentrations of ThEA in negative decadic molar concentrations. Statistical significance was estimated using Student’s *t*-test. *first significant difference (*p*<0.05) vs. 0.3 µM ThEA. Numbers in brackets indicate number of experiments. Ctr, pre-drug value

For comparison with the work of others in the human atrium (Guo et al. 1984), we likewise tested ThEA in H_1_-TG. ThEA has the advantage over histamine that it acts more potently at histamine H_1_-receptors than at other histamine receptors (Neumann et al. 2023). Moreover, in contrast to histamine, ThEA is practically not stimulating histamine H_3_- and histamine H_4_-receptors (Neumann et al. 2023). With increasing concentrations in a cumulative manner, ThEA may initially stimulate histamine H_1_-receptors and then stimulate histamine H_2_-receptors only at higher concentrations. Like histamine itself, any inotropic effect of ThEA is lacking in WT (Fig. 4A). Like histamine, ThEA exerted a transient negative inotropic effect and a sustained positive inotropic in left atrial preparations from H_1_-TG (Fig. 4B). This suggests that both the positive inotropic and the negative inotropic effects of ThEA are mediated via cardiac histamine H_1_-receptors under our experimental conditions. This finding could be validated by the additional presence of the antagonists mepyramine (Fig. 4C) and cimetidine (Fig. 4D) alone or in combination (Fig. 4E). The biphasic effect of ThEA could be abolished by the histamine H_1_-receptor specific antagonist mepyramine (Fig. 4C), whereas the histamine H_2_-receptor specific antagonist cimetidine showed no effects on the inotropy of ThEA (Fig. 4D). We also plotted the positive inotropic effect of ThEA on left atrial preparations of H_1_-TG in Fig. 4G. At 10 µM ThEA, a PIE reached significance. Several such contraction experiments are summarized in Fig. 4F and Fig. 4H. There was a tendency to an increase in force and the first derivative of force versus time by ThEA.

### Contractions in human atrium

**Fig. 5:**
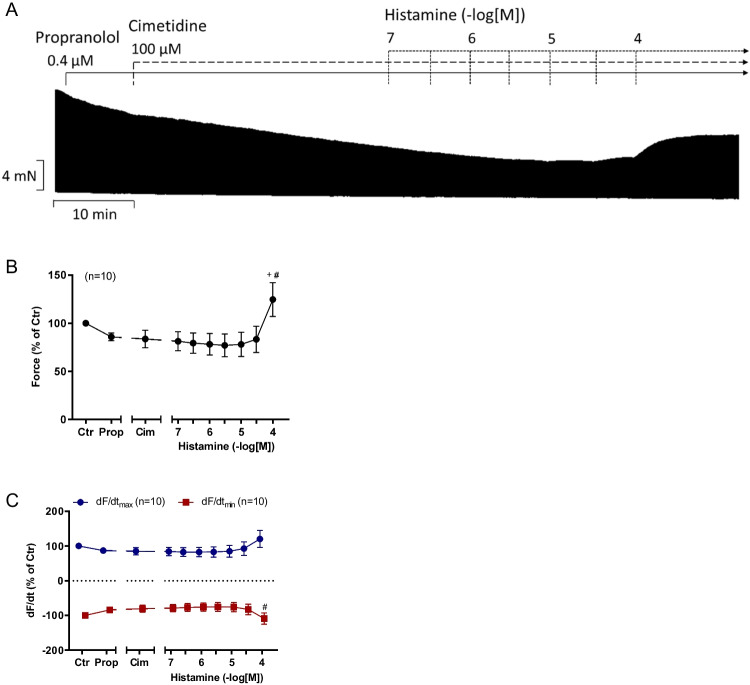
Effects of histamine in human right atrial preparations (HAP) in the presence of cimetidine. Histamine induced a concentration-dependent positive inotropic effect in HAP. Original recording of force of contraction in electrically stimulated (1 Hz) human right atrial muscle strips in the presence of 0.4 µM propranolol and the histamine H_2_-receptor antagonist cimetidine (Figure 5A). Horizontal bar indicates time axis in minutes (min). **B** Force of contraction in % of pre-drug value. **C** Rate of tension development (dF/dt_max_) and rate of tension relaxation (dF/dt_min_) in % of pre-drug value. Ordinate in **A**: Force of contraction in milli Newton (mN). Abscissae in **B** and **C** indicate concentrations of histamine in negative decadic molar concentrations. Statistical significance was estimated using Student’s *t*-test. ^+^first significant difference (*p*<0.05) vs. propranolol, #first significant difference (*p*<0.05) vs. cimetidine. Numbers in brackets indicate number of experiments. Cim, cimetidine; Ctr, pre-drug value; Prop, propranolol

Next, we examine the effect of histamine on force of contraction in HAP in a similar protocol to that in mouse left atria. As seen in the original recording in Fig. 5, histamine exerted a concentration- and time-dependent positive inotropic effect (Fig. 5A). It is apparent in the original recording that no sustained negative inotropic effect, not even a transient negative inotropic effect, occurred (Fig. 5A). Several such experiments are summarized in Fig. 5B for force of contraction and in Fig. 5C for the first derivative of force versus time.

**Fig. 6:**
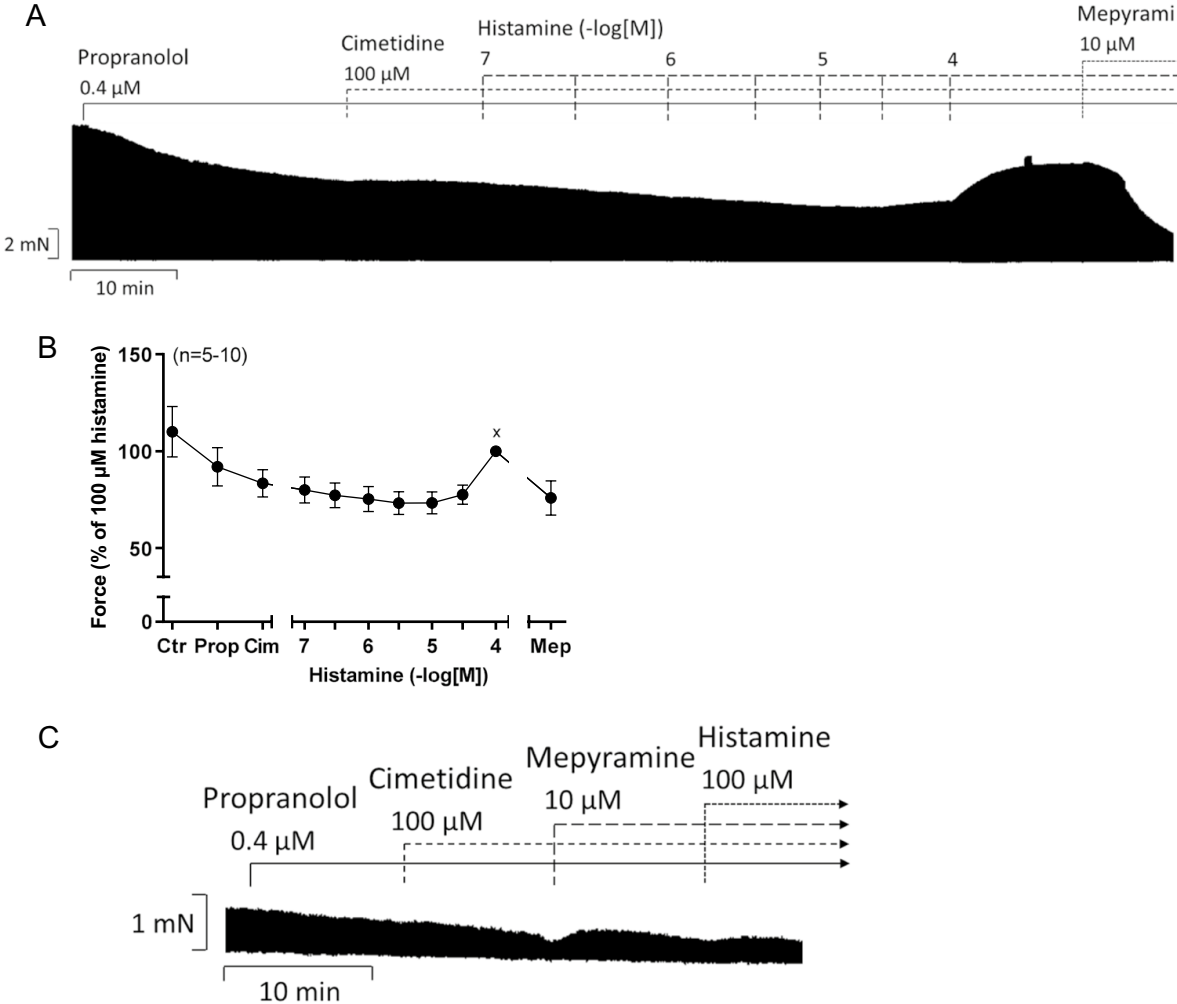
Effects of histamine in HAP in the additional presence of the antagonists mepyramine and cimetidine. The histamine H_1_-receptor antagonist mepyramine could attenuate histamine effects in human right atrial preparations. Original recording of force of contraction in electrically stimulated (1 Hz) human right atrial muscle strips in the presence of 0.4 µM propranolol, the histamine H_2_-receptor antagonist cimetidine (100 µM) and mepyramine (10 µM), which has been added after cumulatively applied histamine (**A**). **C** An original trace of force of contraction in HAP in the presence of 0.4 µM propranolol, the antagonists cimetidine (100 µM) and mepyramine (10 µM) with 100 µM histamine. Horizontal bar in **A** and **C** indicate time axis in minutes (min). **B** Force of contraction in % of 100 µM histamine. Abscissa in **B** indicates concentrations of histamine in negative decadic molar concentrations. Statistical significance was estimated using Student’s *t*-test. ^x^Significant difference (*p*<0.05) vs. mepyramine. Numbers in brackets indicate number of experiments. Cim, cimetidine; Ctr, pre-drug value; Mep, mepyramine; Prop, propranolol

To be sure that the positive inotropic effect is histamine H_1_-receptor mediated, we conducted additional experiments in which we added 10 µM mepyramine after cumulatively applied histamine (Fig. 6A) or before 100 µM histamine (Fig. 6C). This led to a reduction in the histamine effect, which is summarized in Fig. 6B.

**Fig. 7:**
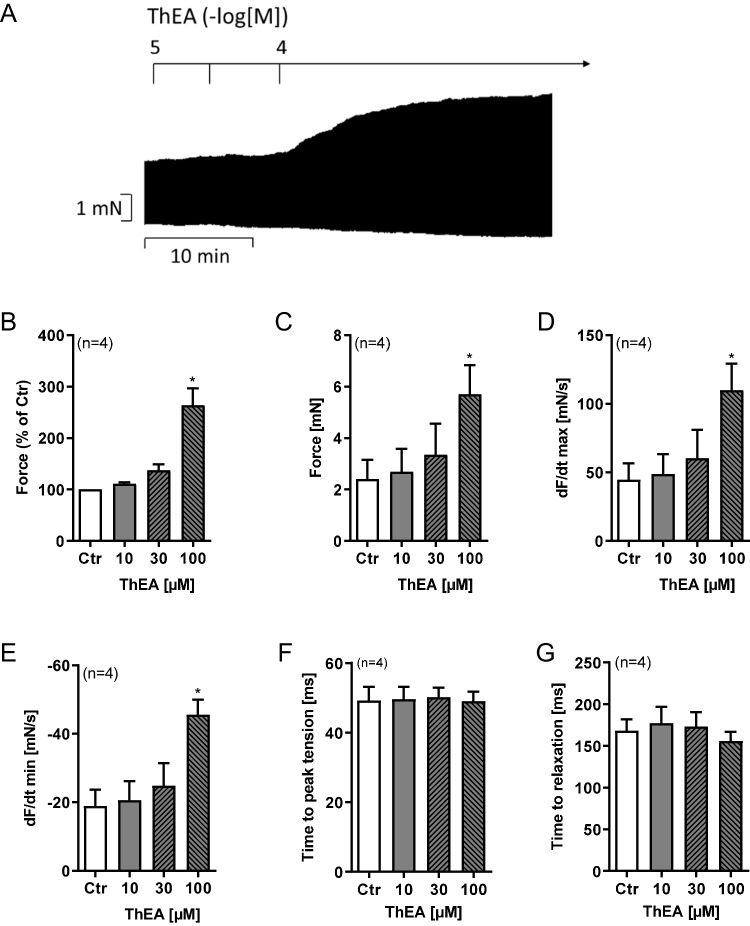
Effects of ThEA in HAP. ThEA induced a concentration- and time-dependent positive inotropic effect in HAP. Original recording of force of contraction in electrically stimulated human right atrial muscle strips (**A**). Horizontal bar indicates time axis in minutes (min). **B** Force of contraction in % of pre-drug value, **C** force of contraction in mN, **D** rate of contraction (dF/dt_max_), **E** rate of relaxation (dF/dt_min_), **F** time to peak tension, **G** time to relaxation. Ordinates in **A** and **C**: Force of contraction in milli Newton (mN). Ordinates in **D** and **E**: Rate of contraction and rate of relaxation in mN per seconds (mN/s). Ordinates in **F** and **G**: Time to peak tension and time to relaxation in milliseconds (ms). Abscissae in **B**, **C**, **D**, **E**, **F**, and indicate concentrations of ThEA in µM. Statistical significance was estimated using Student’s *t*-test. *First significant difference (*p*<0.05) vs. pre-drug value. Numbers in brackets indicate number of experiments. Ctr, pre-drug value

Next we used ThEA instead of histamine in HAP. In a first series of experiments, we gave cumulatively ThEA to HAP. This led to time- and concentration-dependent positive inotropic effects (original recording, Fig. 7A). Several such experiments are summarized for force of contraction in % of pre-drug value (Fig. 7B), force in milli Newton (mN, Fig. 7C), for the maximum or minimum first derivate of force versus time (Fig. 7D and E). Time to peak tension or time of relaxation was not altered in these HAP by ThEA (Fig. 7F and G). Under these conditions, we would have studied histamine H_1_- and histamine H_2_-receptor-mediated effects.

**Fig. 8:**
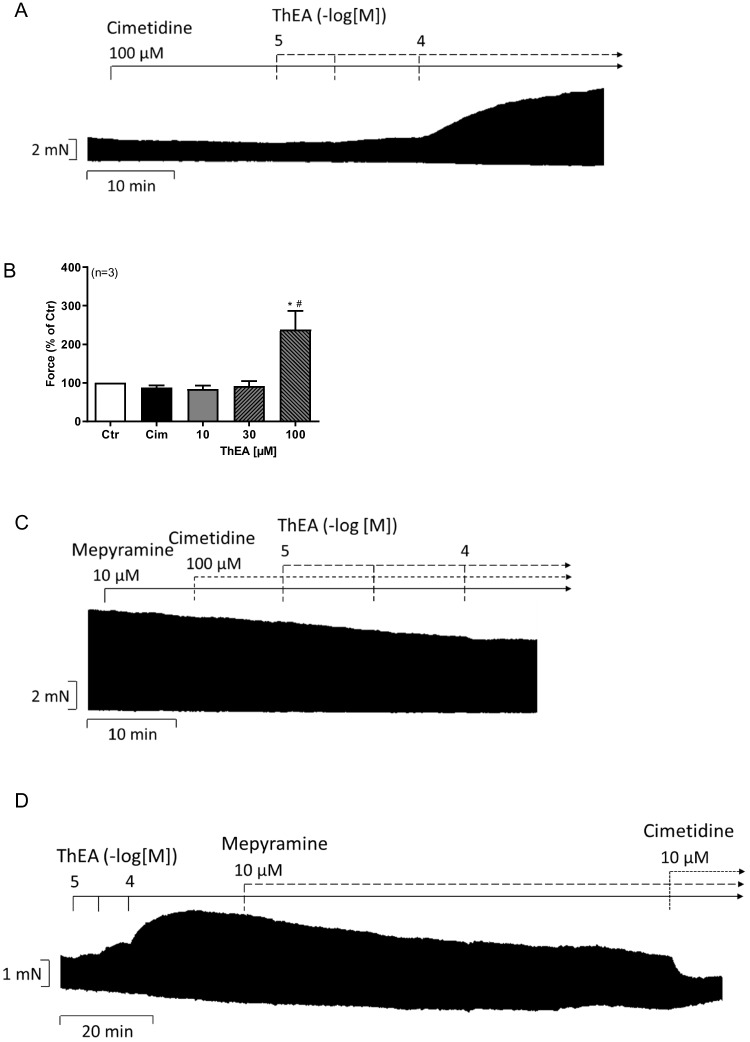

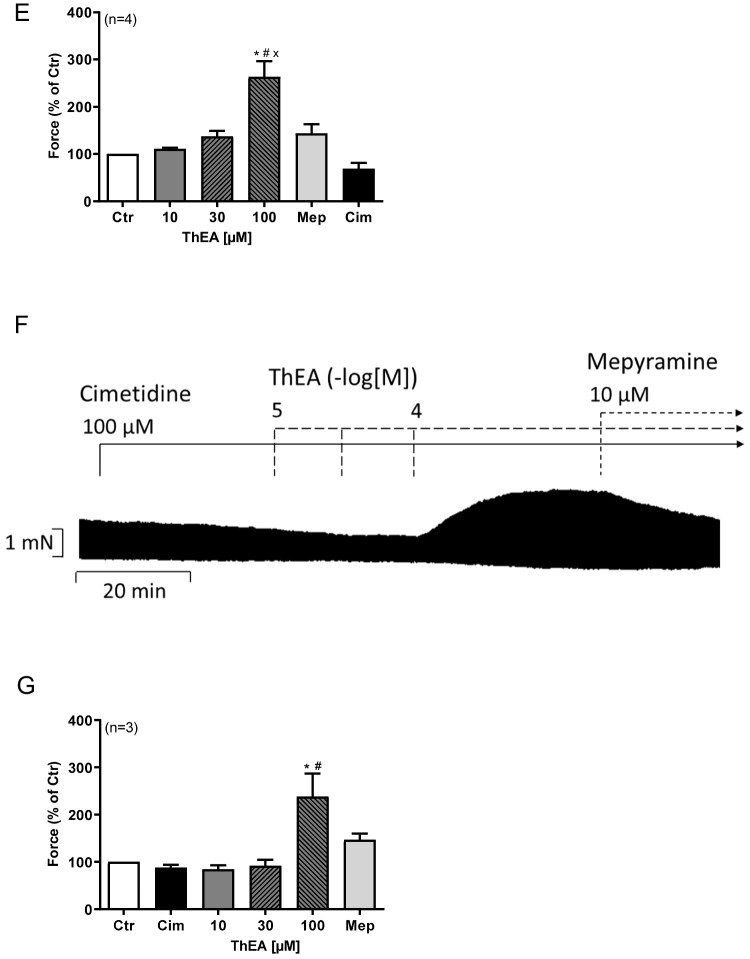
Effects of ThEA in HAP in the presence of cimetidine alone and in different combinations with mepyramine. Original recording of the concentration- and time-dependent positive inotropic effect of ThEA in electrically stimulated human right atrial muscle strips in the presence of the histamine H_2_-receptor antagonist cimetidine alone (**A**), in combination with the histamine H_1_-receptor antagonist mepyramine (**C**), with both antagonists after cumulatively applied ThEA (**D**) and with cimetidine before and mepyramine after ThEA application (**F**). Horizontal bars indicate time axis in minutes (min). **B** Force of contraction in % of control value with previously added cimetidine. **E** Force of contraction in % of control value with mepyramine and cimetidine added after cumulatively applied ThEA. **G** Force of contraction in % of the control value with the addition of cimetidine before and mepyramine after ThEA application. Ordinate in **A**, **C**, **D,** and **F**: Force of contraction in milli Newton (mN). Abscissae in **B**, **E**, and **G** indicate concentrations of ThEA in µM. Statistical significance was estimated using the Student’s *t*-test. *significant difference (*p*<0.05) vs. control, ^#^significant difference (*p*<0.05) vs. cimetidine, ^x^significant difference(*p*<0.05) vs. mepyramine. Numbers in brackets indicate number of experiments. Cim, cimetidine; Ctr, pre-drug value; Mep, mepyramine

To discern these effects, we performed a new series of experiments wherein we first added a high concentration of cimetidine to block all histamine H_2_-receptors. Then we added again ThEA and noted time- and concentration-dependent PIE, which we regard as histamine H_1_-receptor mediated (Fig. 8A). Such data are summarized in Fig. 8B. Next we added both antagonists, first mepyramine as histamine H_1_- and then cimetidine as histamine H_2_-receptor antagonist before (Fig. 8C) and after cumulatively applied ThEA (Fig. 8D). Such data were summarized in Fig. 8E. We also performed experiments in which we blocked all histamine H_2_-receptors with cimetidine first and then added mepyramine after ThEA increased force of contraction (Fig. 8F, Fig. 8G).

**Fig. 9:**
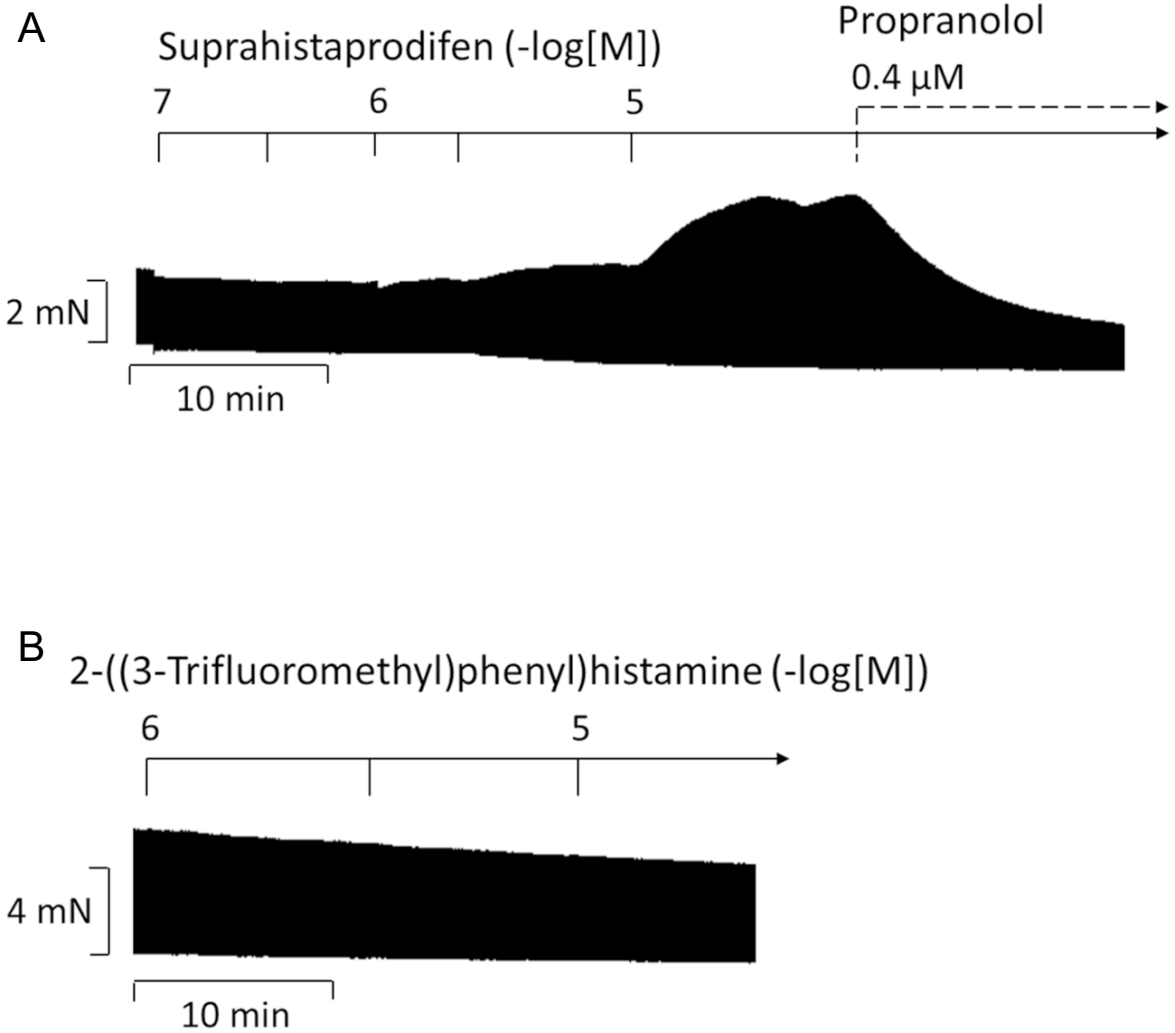
Original recordings of HAP with the agonists suprahistaprodifen and 2-(3-trifluoromethylphenyl)histamine. Original recordings of a concentration-response curve of suprahistaprodifen (**A**) and 2-(3-trifluoromethylphenyl)histamine (**B**) in milli Newton (mN) in electrically stimulated (1 Hz) human right atrial muscle strips. Horizontal bar indicates time axis in minutes (min). Suprahistaprodifen showed a positive inotropic effect, which was abolished by the β-adrenergic receptor antagonist propranolol. 2-(3-Trifluoromethylphenyl)histamine up to 10 µM failed to increase force of contraction in HAP

Finally, it had been reported that suprahistaprodifen in cell cultures was a very potent agonist, perhaps the most potent and selective agonist at histamine H_1_-receptors (Carman-Krzan et al. 2003). However, it was never studied in the human heart. We noted a clear positive inotropic effect to suprahistaprodifen in HAP (Fig. 9A). However, this effect was antagonized by propranolol and our interpretation is that suprahistaprodifen is not a useful histamine H_1_-receptor agonist in the human heart; perhaps, it releases intracardiac noradrenaline (Supplementary Data 2A, Data 2B and Data 2C). Another histamine H_1_-receptor agonist is 2-(3-trifluoromethylphenyl) histamine. It was never studied in human cardiac tissue. However, in contrast to suprahistaprodifen, 2-(3-trifluoromethylphenyl)histamine up to 10 µM failed to increase force of contraction in HAP (Fig. 9B).

## Discussion

### Main new findings

The main new finding in our study is that stimulation of histamine H_1_-receptors can directly exert a positive inotropic effect in HAP without any negative inotropic effect under our experimental conditions.

Moreover, we show under the same experimental conditions in parallel experiments with stimulation of the histamine H_1_-receptors, a positive inotropic effect was observed in the left atria from H_1_-TG. Interestingly, the histamine H_1_-receptor-mediated positive inotropic effect was always preceded by a transient negative inotropic effect which is also mediated by histamine H_1_-receptors. From this, we concluded that in principle, the positive and the negative inotropic effects are histamine H_1_-receptors mediated. These data are in line with those of others in guinea pig hearts: in ventricular guinea pig preparations, when histamine H_2_-receptors were blocked by an antagonist, histamine H_1_-receptors mediated a negative inotropic effect (Zavecz and Levi 1978). Thus, in the guinea-pig ventricle, the phenotype of the effect of histamine at first glance is similar to that in H_1_-TG in the left atrium. In contrast to the guinea pig ventricle, in the guinea pig left atrium, histamine exerts a positive inotropic effect which is entirely mediated via histamine H_1_-receptors. This clearly suggests that for the potential effect of the histamine H_1_-receptor in the heart, the signal transduction system must be decisive: it must be different in the left atrium compared to the left ventricle of the guinea pig. This signal transduction difference needs to be elucidated in future studies. Comparing mice and men, our data argue that species differences in atrial preparations occur. In WT mice, no inotropic effect to histamine is noted, whereas the mRNA for the histamine H_1_-receptors and the histamine H_2_-receptor is expressed (Gergs et al. 2019). When human histamine H_1_-receptors are expressed in mice (H_1_-TG), then they are not silent anymore but exert in the same preparation opposite effects: a decrease and over time an increase in force of contraction is noted. When one looks at human atrium, where physiologically the mRNA of the human histamine H_1_-receptor is expressed, only a positive inotropic effect to histamine via histamine H_1_-receptors is noted. Hence, something might impair the negative inotropic effect in the human heart. This might be the histamine H_2_-receptor that may be constitutively active, or heterodimeric histamine H_1_- and histamine H_2_-receptors (that have been observed in some cells: Neumann et al. 2023) that have altered signaling or simply the inhibitory signal transduction system for the histamine H_1_-receptor is lacking in human atrial cardiomyocytes, while it is present in mouse atrial cardiomyocytes. Currently, these explanations are hypothetical and may all be valid to some degree and should be subject to further research.

As concerns the mechanism of the positive inotropic effect of the stimulation of the histamine H_1_-receptor in the human heart, it is instructive to discuss our present knowledge from animal experiments mainly in rabbit and guinea pig atria. Here others reported the second messenger might be inositoltrisphosphate (IP3) because IP3 increases after simulation of the histamine H_1_-receptor in the rabbit atrium (Hattori et al. 1990). However, IP3 might not be critically involved in the PIE of histamine H_1_-receptor stimulation (Hattori et al. 1989). If nevertheless phospholipase C is involved, it might inhibit cardiac phosphatases which would increase force of contraction (Herzig and Neumann 2000). In other studies, a histamine H_1_-receptor stimulation increased cGMP (Richelson 1978, Hattori et al. 1988a, Hattori et al. 1990). Moreover, histamine H_1_-receptor stimulation could activate tyrosine kinase and this led to protein phosphorylation. One has suggested that this phosphorylation might led to enhanced Ca^2+^ sensitivity of myofilaments and thus might increase force of contraction (Akaishi et al. 2000). The histamine H_1_-receptors might also couple to ion channels, e.g., in guinea pig left atrial preparations (Leurs et al. 1994, Matsumoto et al. 1999, Wang and Kotlikoff 2000). At least in guinea pig left atrium, the PIE of histamine H_1_-receptor stimulation seems to be mediated in part by Ca^2+^ released from the sarcoplasmic reticulum and in part by Ca^2+^ passing through the L-type calcium channel (Hattori et al. 1988b, review: Hattori et al. 2017). These mechanisms are in part plausible and may act in concert. We have discussed this issue in detail elsewhere (Neumann et al. 2023). Thus, it needs to be elucidated which of the described mechanisms of a positive inotropic effect through histamine H_1_-receptors is used in the human heart.

A limitation of the present study lies in the fact that we cannot compare directly across species the expression of the histamine H_1_-receptors. More specifically, it would be mechanistically important to know how the protein levels of the histamine H_1_-receptor are different between H_1_-TG and the human atrium. In other words, one would like to know whether H_1_-TG express the same protein level of histamine H_1_-receptor as the human atrium. This would be helpful for future works with the H_1_-TG. However, this question cannot be answered presently. As reported here with a commercially available antibody, we failed to detect the human histamine H_1_-receptor even in H_1_-TG where high levels of this protein is expected. Moreover, this comparison between mouse and man is also with our current protocols not possible with PCR because good primers for the housekeeping gene HPRT1 in human atrium did not work in mouse heart. In the past, we used PCR with success within mouse: we reported that we detected a high expression of the mRNA of the histamine H_1_-receptor normalized to the house keeping gene Hprt1 (Rayo Abella et al. 2024). Hence, regrettably at present, we cannot provide protein expression levels of histamine H_1_-receptors between human and mouse atrium and one has to attend that appropriate antibodies become commercially available in the future.

Under the same experimental conditions as in H_1_-TG, we failed to see a negative inotropic effect of histamine in the human atrium. In contrast, we and others before us (Neumann et al. 2023) detected a positive inotropic effect to histamine. This positive inotropic effect of histamine is cimetidine sensitive and on that basis is thought to be mainly or exclusively histamine H_2_-receptors mediated (Ginsburg et al. 1980, Baumann et al. 1984). The new experimental finding of the present work is that even when we blocked histamine H_2_-receptors with cimetidine, we still noticed an additional positive inotropic effect to stimulation of the histamine H_1_-receptor which was blocked by mepyramine and which we therefore regard as histamine H_1_-receptor mediated in the human atrium. Others presented the opposite finding, namely solely a negative inotropic effect in human atrial preparations via histamine H_1_-receptors (Guo et al. 1984). In order to understand why we got opposite results, we tried to emulate their experimental conditions but failed to reproduce their data. Those authors (Guo et al. 1984) employed ThEA as agonist for histamine H_1_-receptors. With ThEA, we could detect consistently and reproducibly a positive inotropic effect in HAP. In line with earlier work in guinea pig heart and human atrial preparations, the effects of ThEA were in part sensitive to cimetidine and in part sensitive to mepyramine suggesting that ThEA acted at both histamine H_1_-receptors and histamine H_2_-receptors. However, in contrast to findings in guinea pig ventricle (which contains functionally about equal amounts of histamine H_1_-receptors and histamine H_2_-receptors, Zavecz and Levi 1978) or human atrium (Guo et all. 1984), in our studies, we only noted positive inotropic effects to ThEA in HAP_._

Therefore, we deemed it instructive to also study ThEA in H_1_-TG which we had not studied in our initial report on the generation of H_1_-TG (Rayo Abella et al. 2024) before. In LA from H_1_-TG, we detected transient negative inotropic effects of ThEA, which were never detected in human atrium. Thus, with histamine or ThEA as agonists, we could induce in principle in the isolated mammalian atrium under our experimental conditions a negative inotropic effect, namely in left atrial preparations from H_1_-TG. Hence, one cannot easily argue that we have overlooked for methodological reasons a negative inotropic effect via histamine H_1_-receptor stimulation in the human atrium. If a negative inotropic effect exists, we can measure that.

Ginsburg et al. (1980) detected with ThEA (300 µM) a positive inotropic effect that was not blocked by 1 µM pyrobutamine, an early histamine H_1_-receptor antagonist, but the positive inotropic effect to ThEA was blocked by 10 µM cimetidine and was thus regarded as histamine H_2_-receptor mediated (Ginsburg et al. 1980). We would like to point out that Ginsburg et al. 1980 studied using HAP from very sick patients; their samples originated from recipients of heart transplantations. This might explain in part their lack of detection of histamine H_1_-receptor-mediated positive inotropic effects. Hence, one hypothesis might be that like for β_1_-adrenoceptor-mediated positive inotropic effects, also histamine H_1_-receptor-mediated positive inotropic effects are reduced or absent when the heart failure progresses in a patient (Port and Bristow 2001). This hypothesis needs to be tested in future studies.

Regrettably, no good selective histamine H_1_-receptor agonist is currently available. Sometimes suprahistaprodifen is recommended as histamine H_1_-receptor agonist (Carman-Krzan et al. 2003). In our hands, suprahistaprodifen exerts positive inotropic effect in HAP. However, this was propranolol sensitive and thus was not mediated via histamine H_1_-receptors. One explanation for our findings would be that suprahistaprodifen acted like an indirect sympathomimetic agent and released noradrenaline from nerve cells in the human atrium. The liberated noradrenaline then stimulates β-adrenoceptors. We have recently shown for amphetamine, methamphetamine, or cathine that they increase force of contraction in the human heart as indirect sympathomimetic agents under our experimental conditions in the human atrium (Neumann et al. 2023). Therefore, such a mechanism might exist also for suprahistaprodifen. However, suprahistaprodifen is not useful to study histamine H_1_-receptor in contraction studies in multicellular preparations of the human heart. Conceivably, in isolated human atrial cardiomyocytes where endogenous noradrenaline from nerve cells is lacking, suprahistaprodifen may be useful, but that is currently hypothetical. We used as an alternative drug to stimulate human histamine H_1_-receptors a compound called 2-(3-trifluoromethylphenyl)histamine (Leschke et al. 1995). However, 2-(3-trifluoromethylphenyl)histamine seemed in cell culture to couple differently to second messengers than histamine (Lieb et al. 2016). 2-(3-Trifluoromethylphenyl)histamine was devoid of any inotropic effect in human atrium, suggesting that the stimulation of histamine H_1_-receptors or signal transduction is critically agonist dependent in the human heart. Apparently, one has to use histamine or ThEA in human atrium to stimulate histamine H_1_-receptors.

Some support for our data comes from studies on human cardiac stem cells, where the authors found evidence for the presence and functional role of histamine H_1_-receptors in these cells (Ferreira-Martins et al. 2009, Zhu et al. 2020).

We have not studied here in depth the signal transduction of the human histamine H_1_-receptor because that was beyond the scope of the present paper.

### Limitations of the study

We have no access to human ventricular preparations. In human atrial preparations, not only histamine H_1_-receptor but also histamine H_2_-receptors are present. In H_1_-TG, only histamine H_1_-receptor are overexpressed and are functional in H_1_-TG but not in wild-type mice. Thus, our mouse model does not fully reflect the clinical expression pattern of histamine receptors in the human heart especially the human atrium. Moreover, we cannot directly compare the expression of the histamine-H_1_-receptor between H_1_-TG and human atrium. This was not possible on protein level due to lack of a commercial antibody that detects the histamine H_1_-receptor in Western blots (Supplementary Data 1). Furthermore, PCR data for the mRNA of histamine H_1_-receptor are of limited value because the protein of the histamine H_1_-receptor is responsible for its contractile effects and not the mRNA. Direct comparison of mRNA data between mouse and man is also not really possible by qPCR for lack of a common denominator that is stable between species.

In summary, we detected both a positive and a negative inotropic effect of histamine in LA in H_1_-TG. In human atrial preparations, however, we only noted a positive inotropic effect when we stimulated histamine H_1_-receptor with two different agonists.

## Supplementary Information

Below is the link to the electronic supplementary material.

Supplementary file1 (PDF 139 KB)

## Data Availability

All source data for this work (or generated in this study) are available upon reasonable request.
