# Supplementary material for: Stimulation of histamine H1-receptors produces a positive inotropic effect in the human atrium
Source: Naunyn Schmiedebergs Arch Pharmacol. 2024 Dec 27;398(6):7235–50. doi: 10.1007/s00210-024-03735-y (PMC12125156; doi:10.1007/s00210-024-03735-y)

Supplementary Data

Data 1

HRH$_1$-AB: antibodies, ABIN719606 (AI12043764), dilution 1:500; 20 µg loaded protein

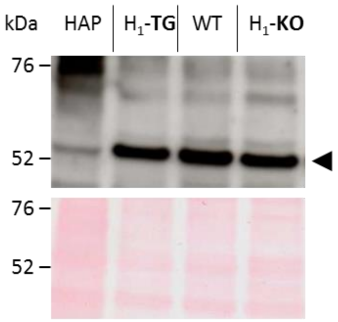

Data 2

2A WT

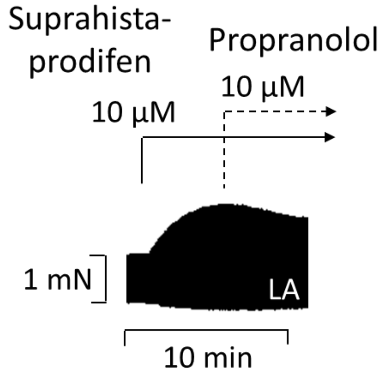

2B WT

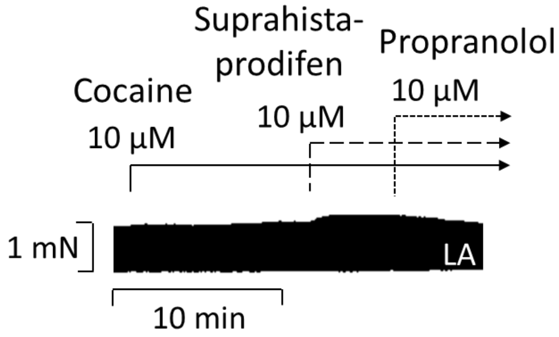

2C

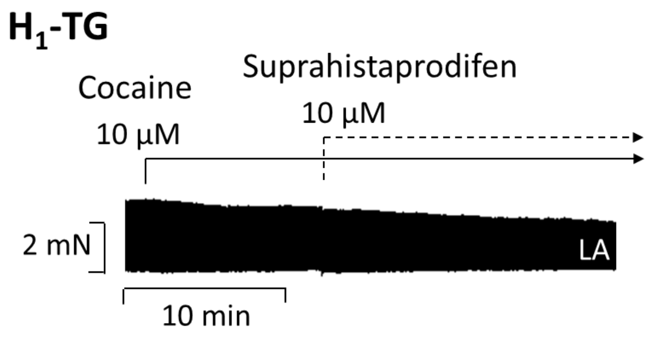

Supplement: Supplementary file 1 — Supplementary file1 (PDF 139 KB) [file 210_2024_3735_MOESM1_ESM.pdf]
